# Activities of Amphioxus GH-Like Protein in Osmoregulation: Insight into Origin of Vertebrate GH Family

**DOI:** 10.1155/2017/9538685

**Published:** 2017-03-17

**Authors:** Mengyang Li, Chengyan Jiang, Yu Zhang, Shicui Zhang

**Affiliations:** ^1^Laboratory for Evolution & Development, Institute of Evolution & Marine Biodiversity and Department of Marine Biology, Ocean University of China, Qingdao 266003, China; ^2^Institute for Translational Medicine, Qingdao University, Qingdao 266021, China; ^3^College of Life Science and Technology, Hong He University, Mengzi, Yunnan 661100, China

## Abstract

GH is known to play an important role in both growth promotion and osmoregulation in vertebrates. We have shown that amphioxus possesses a single GH-like hormone (GHl) gene encoding a functional protein capable of promoting growth. However, if GHl can mediate osmoregulation remains open. Here, we demonstrated clearly that GHl increased not only the survival rate of amphioxus but also the muscle moisture under high salinity. Moreover, GHl induced the expression of both the ion transporter Na^+^-K^+^-ATPase (NKA) and Na^+^-K^+^-2Cl^−^ cotransporter (NKCC) in the gill as well as the mediator of GH action IGFl in the hepatic caecum, indicating that GHl fulfills this osmoregulatory activity through the same mechanisms of vertebrate GH. These results together suggest that the osmoregulatory activities of GH had emerged in the basal chordate amphioxus. We also proposed a new model depicting the origin of pituitary hormone family in vertebrates.

## 1. Introduction

Growth hormone (GH) and prolactin (PRL) are structurally related pituitary polypeptide hormones that belong to a superfamily of helical cytokines. Both GH and PRL act by interacting with single transmembrane domain receptors that are also structurally related and belong to the type 1 cytokine receptor superfamily [1, 2]. These hormones and their receptors are believed to have evolved from common ancestral genes through gene duplication and subsequent divergence early in vertebrate evolution [3, 4]. However, questions remain about the timing and subsequent elaboration of gene duplication and the elucidation of genetic innovations that may have contributed to the origin and subsequent divergence of pituitary hormones and their receptors in modern vertebrates.

GH and PRL are both multifunctional and share some overlapping biological properties [4, 5]. For example, they are both known to be involved in the regulation of hydromineral balance in fishes [6, 7]. GH has been shown to facilitate seawater (SW) adaptation in several fishes including salmonids, tilapia, and killifish [8–11], while PRL shown to be an important freshwater- (FW-) adapting hormone regulating FW adaptation in tilapia and killifish [12, 13]. GH has been reported to be able to induce the production of ion transporter Na^+^-K^+^-ATPase (NKA) [11, 14, 15] and Na^+^-K^+^-2Cl^−^ cotransporter (NKCC) [16] that provide the driving force for ion-transporting functions of chloride cells in the gills [17–19]. In addition, insulin-like growth factor-I (IGF-I), which mediates many growth-promoting actions of GH, also appears to mediate the osmoregulatory activity of GH during SW acclimation in salmonids [20, 21]. By contrast, PRL usually maintains plasma homeostasis of fishes in FW by altering salt and water permeability across epithelial cell membranes in the gill, gut, and kidney [22–25].

Amphioxus or lancelet belongs to the subphylum Cephalochordata, an extant representative of the most basal chordates. Recently, we have found that amphioxus possesses a single GH-like hormone (GHl) gene encoding a functional protein capable of promoting growth [26]. Moreover, the animal also has a homologue GH/PRLlBP of vertebrate GH-binding protein (GHBP), which is a soluble and truncated form of GHR lacking transmembrane and intercellular parts [26]. However, does GHl, like vertebrate GH, play a role in osmoregulation? And if so, how does it function in amphioxus? The aim of this study is therefore to answer these questions.

## 2. Materials and Methods

### 2.1. General Experimental Design

In this study, we first injected the amphioxus with recombinant GHl to test if GHl plays an osmoregulatory role as vertebrate GH does. We then explored the osmoregulatory mechanism of GHl. Finally, we investigated if a vertebrate-like GH/IGF axis is also involved in the osmoregulation of amphioxus by examining the correlation of salinity and expression of GHl/IGFl axis genes.

### 2.2. Animals

Animal experiments were approved by the Ethics Committee of the Laboratory Animal Administration of Shandong Province (permission number SD2007695). All the experiments were performed in accordance with relevant guidelines and regulations. Amphioxus *Branchiostoma japonicum* with average body length of about 2 cm were collected from the seashore in Qingdao city, Shandong province, China. They were fed and cultured as described by Wang and Zhang [27].

### 2.3. Recombinant Proteins

The recombinant protein of amphioxus GHl (rGHl) was prepared as described by Li et al. [26]. Zebrafish recombinant GH (rzGH) was purchased from ProSpec (East Brunswick, NJ, USA). The recombinant protein of zebrafish PRL (rzPRL) was produced by the methods of Li et al. [26]. Briefly, the cDNA encoding mature PRL (25 to 210 amino acids) was amplified by PCR, and the PCR products were digested with *EcorI* and *XhoI* and subcloned into the plasmid expression vector pET28a (Novagen, Germany) cut with the same restriction enzymes. The plasmid constructed was verified by sequencing and transformed into the cells of *Escherichia coli* BL21 (DE3). The recombinant protein was expressed, purified, and refolded as described by Li et al. [26]. The purified protein was analyzed on a 12% SDS-PAGE gel and immunostained using anti-His-tag monoclonal antibody (CWBIO, China) as the primary antibody. All the protein concentrations were determined with BCA protein assay kit (Beyotime, China).

### 2.4. Salinity Tolerance Assay

Salinity tolerance assay was performed to explore if amphioxus GHl, like fish GH or PRL, plays an osmoregulatory role. Pilot experiments showed that when amphioxus was administered with 10 *μ*l of saline (0.9% NaCl) by intracoelomic injection and cultured in seawater with 38‰ or 15‰ salinity, 80% to 100% mortality was observed at 96 h after injection (data not shown). Therefore, these salinities were chosen for the following experiments. Amphioxi were first acclimated to 30‰ seawater, and then, 20 animals per group were each injected with either 10 *μ*l of saline or 10 *μ*l saline containing 10 ng rGHl or 10 ng rzGH or 10 ng rzPRL. The dosages used were according to the previous studies in fishes [14, 16, 28]. Soon after injection, the animals were cultured in 500 ml of seawater with 38‰ or 15‰ salinity at ambient photoperiod and at room temperature. The salinity was prepared by mixing Millipore ultrapure water with artificial sea salt (Haijia, China). The seawater was changed every 24 h, and the mortality was checked simultaneously. The beating of velar tentacles on the velum can be used as “living marker” of amphioxus; if the tentacles stopped beating, even in response to pricking, then the animals were believed to be “dead.”

### 2.5. Cloning and Expression of *nka* and *nkcc*

Na^+^-K^+^-ATPase (NKA) and Na^+^-K^+^-2Cl^−^ cotransporter (NKCC) located in the basolateral membrane of the chloride cells in the gill of fishes are accepted as the major ion transporters [17–19]. Our searching of *B. floridae* genome database (http://genome.jgi-psf.org//Brafl1/Brafl1.home.html) for the homologues of vertebrate NKA and NKCC (using NKA alpha 1 and NKCC1 as queries) revealed the presence of a single NKA (GenBank accession number: XP_002610679.1) and NKCC (GenBank accession number: XP_002609755.1), respectively. We then set to clone *nka* and *nkcc* from *B. japonicum.* Total RNAs were extracted with Trizol (TaKaRa, Dalian, China) from *B. japonicum* and digested with RNase-free DNase to eliminate the genomic contamination. The first-strand cDNA was synthesized with reverse transcription system using oligo d(T) primer. To amplify the fragments of *nka* and *nkcc* cDNAs, polymerase chain reaction (PCR) was performed using the first-strand cDNA as template, in a total volume of 20 *μ*l PCR reaction mixture containing 1 × PCR buffer, 0.5 unit of EX Taq DNA polymerase, and 0.4 *μ*M of the gene-specific primers P5 and P6 as well as P9 and P10 (Table 1), which were designed on the basis of the putative *nka* and *nkcc* sequences found in *B. floridae* genome database. PCR was carried out at 94°C for 5 min, followed by 34 cycles at 94°C for 30 s, 54°C for 30 s, 72°C for 1 min, and a final extension step at 72°C for 7 min. The PCR products were gel-purified using DNA gel extraction kit (Axygen, Union City, USA), ligated into the T/A cloning vector pGEM-T easy (Promega, Shanghai, China) at 4°C overnight, and transformed into the competent cells of *E. coli* (Tiangen, Beijing, China). The positive clones were selected and sequenced with ABI PRISM 3730 DNA sequencer. The sequences were searched in GenBank with BLASTx for comparative analysis. Sequence comparison against NKA and NKCC was performed by the MegAlign program of the LASERGENE software suit (DNASTAR). Protein domains were analyzed using the SMART program (http://smart.embl-heidelberg.de/).

To examine the expression profiles of *nka* and *nkcc*, whole amphioxus and the different tissues including the gill, hindgut, hepatic caecum, notochord, muscle, and skin dissected out of amphioxus were homogenized in Trizol Reagent (Invitrogen) and stored at −80°C until use. Total RNAs extraction and the first-strand cDNA synthesis were performed as above. Semiquantitative RT-PCR (qRT-PCR) was performed using quantified cDNA templates (about 0.3 to 0.4 *μ*g/*μ*l) and the primers on ABI 7500 real-time PCR system (Applied Biosystems, USA). The PCR primers specific of *nka* (P7 and P8), *nkcc* (P11 and P12), and *ef1a* (P13 and P14) were designed using premier 5.0 program [29]. The *ef1a* gene was chosen as the reference for internal standardization. SYBR Premix ExTaq™ (Takara, Japan) was used according to the manufacturer's protocol with a primer concentration of 200 nM. The reaction conditions were as follows: 95°C for 1 min, followed by 40 cycles of 95°C for 5 s, 60°C for 15 s, and 72°C for 35 s. Dissociation analysis was performed at the end of each PCR reaction to confirm the amplification specificity. After the PCR program, the data were analyzed with ABI 7500 SDS software (Applied Biosystems) and quantified with the comparative Ct method (2^–ΔΔ^Ct) based on Ct values for genes and the reference *ef1a* in order to calculate the relative mRNA expression level.

### 2.6. Assay for Effects of Salinity on Gene Expression and Muscle Moisture

A total of 60 amphioxi was acclimated in the seawater with 25‰ salinity for 15 days and then divided into 2 groups: 30 animals were transferred to seawater with 30‰ salinity, and 30 still maintained in seawater with 25‰ salinity. Ten animals were then sampled from each group at 0, 48, and 72 h after transferring to seawater, and the Hatschek's pit, gill, and hepatic caecum were dissected out of the animals and homogenized in Trizol. The preparation of total RNAs and the expression of both *nka* and *nkcc* in the gill and *ghl* in the Hatschek's pit and gill as well as *gh/prllbp* and *igfl* in the hepatic caecum and gill were performed as described above. The collection of the Hatschek's pit was carried out as described by Fang and Wang [30]. The wheel organ on the right side of the notochord was the part with Hatschek's pit and was dissected out under microscope. The PCR primers specific of *nka* (P7 and P8) and *nkcc* (P11 and P12) were as above, and the primers specific of *gh/prllbp* (P15 and P16), *igfl* (P17 and P18), and *ghl* (P19 and P20) were designed using premier 5.0 program.

The muscle was also dissected out of the animals sampled. The tissue was cut into pieces 1 mm^3^, dried on filter paper, and soon transferred to 1.5 ml Eppendorf tubes. The muscle moisture was measured as described by Mccormick [28] and Breves et al. [31]. Briefly, 100 mg of the muscle from each group was dried to a constant weight at 60°C, and the water content was then measured gravimetrically.

### 2.7. Assay for Effects of rGHl and rzGH on Gene Expression In Vitro

The gill and hepatic caecum were dissected out of amphioxus, cut into pieces (~1 mm^3^), and cultured in MEM medium containing 2.2 mg/ml NaHCO_3_, 100 IU/ml penicillin, 100 *μ*g/ml streptomycin, and different concentrations of rGHl or rzGH (0, 10, 100, and 1000 ng/ml) at 25°C. After 4 h culture, the tissue pieces of the gill and hepatic caecum were pooled and homogenized immediately in Trizol Reagent, and total RNAs were prepared. The expressions of *nka* and *nkcc* in the gill as well as *gh/prllbp* and *igfl* in the gill and hepatic caecum were analyzed by qRT-PCR as above.

### 2.8. Assay for Effects of rGHl and rzGH on Gene Expression In Vivo

A total of 90 amphioxi was divided into 3 groups (30/group), and each animal was injected with either 10 *μ*l of saline or 10 *μ*l saline plus 10 ng rGHl or 10 ng rzGH. They were then cultured in 500 ml natural seawater and fed once a day with the single-cell alga *Spirulina* sp. Ten amphioxi were sampled from each group at 0, 24, and 48 h after injection, respectively, and the gill and hepatic caecum were dissected out of the animals. Both the tissues were homogenized in Trizol, and total RNAs were prepared. The expressions of *nka* and *nkcc* in the gill as well as *gh/prllbp* and *igfl* in the gill and hepatic caecum were analyzed by qRT-PCR as above.

### 2.9. Assay for Effects of rGHl and rzGH on NKA Activity and Muscle Moisture

A total of 90 amphioxi was divided into 3 groups (30/group), and each animal was injected with either 10 *μ*l of saline or 10 *μ*l saline plus 10 ng rGHl or 10 ng rzGH. They were cultured and sampled as above. Both the gill and muscle were dissected out of the animals sampled. The tissues were cut into pieces 1 mm^3^, dried on filter paper, and soon transferred to precooled 1.5 ml Eppendorf tubes. The NKA activity in the gill (ca. 50 mg per group) was analyzed with NKA activity kit (Solarbio, Beijing) as described as Zeng et al. [32]. One unit of enzymatic activity was defined as 1 *μ*mole ADP released per hour. The muscle moisture was measured as described above.

### 2.10. Statistical Analysis

Salinity tolerance experiment was repeated twice, while all the other experiments were performed in triplicate and repeated three times. The survival curve was generated by GraphPad Prism 5. The data of the experiments of effects of salinity on gene expression and muscle moisture were analyzed by two-way ANOVA, while the other data analyzed by one-way ANOVA. The difference at *p* < 0.05 was considered significant. All the data were expressed as mean ± SEM.

## 3. Results

### 3.1. rGHl Enhances Salinity Tolerance of Amphioxus

Recombinant amphioxus GH-like protein rGHl was expressed and purified as described by Li et al. [26]. Recombinant zebrafish prolactin rzPRL with His tag was expressed in *E. coli* and purified by chromatography on a Ni-NTA resin column. rzPRL was subjected to SDS-PAGE, which yielded a single band corresponding to the expected sizes of ~24.6 kDa. Western blotting revealed that rzPRL reacted with rabbit anti-His-tag antibody, indicating that rzPRL was correctly expressed (see Supplementary Figure 1 in Supplementary Material available online at https://doi.org/10.1155/2017/9538685).

**Figure 1:**
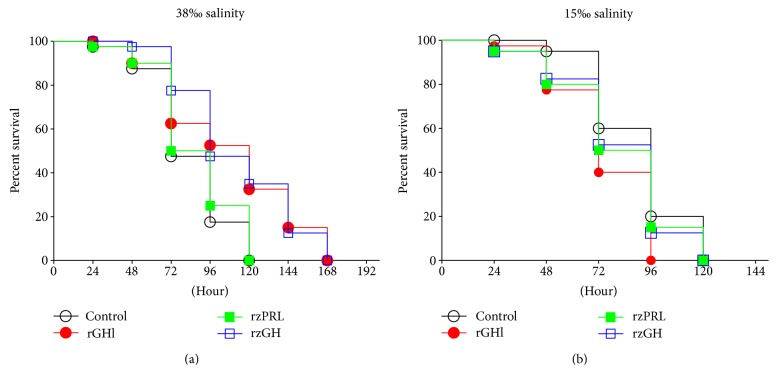
The survival rates of amphioxus. The animals were injected with saline, rGHl, rzGH, or rzPRL by intracoelomic injection, followed by culture in seawater with 38‰ salinity (a) or 15‰ salinity (b). Survival was recorded every 24 h. Data are from two independent experiments.

Figure 1 shows the survival rates of amphioxus injected with saline, rGHl, rzGH, or rzPRL, followed by culture in seawater with different salinity. The culture under high salinity (38‰) resulted in 52.5%, 82.5%, and 100% as well as 50%, 75% and 100% cumulative mortality, respectively, in the saline- and rzPRL-injected amphioxus at 72, 96, and 120 h after injection. The mortalities of these two groups were not statistically different. The same culture caused only 37.5%, 48.5%, and 67.5% as well as 22.5%, 52.5%, and 65% cumulative mortality, separately, in the rGHl- and rzGH-injected animals at the same experimental periods. The survival rates of the rGHl- and rzGH-injected groups were significantly higher than control (Figure 1(a)). By contrast, the culture under low salinity (15‰) resulted in 40% and 80%, 60% and 100%, and 47.5% and 87.5% as well as 50% and 85% cumulative mortality, individually, in the saline-, rGHl-, rzGH-, and rzPRL-injected animals at 72 and 96 h after injection, and none of the animals survived by 120 h. No statistical difference was observed in the groups (Figure 1(b)). These data together indicated that amphioxus GHl, like zebrafish GH, could promote salinity tolerance of amphioxus, but zebrafish PRL could not.

### 3.2. rGHl Induces Expression of *nka* and *nkcc*

**Figure 2:**
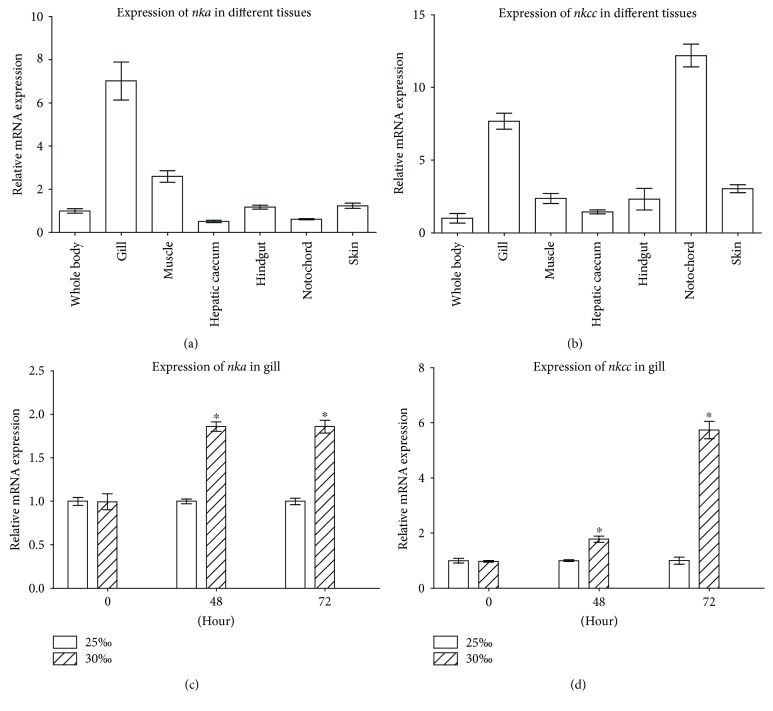
Identification of *nka* and *nkcc*. (a) The expression profiles of *nka* in the different tissues including the gill, hindgut, hepatic caecum, skin, notochord, and muscle. (b) The expression profiles of *nkcc* in the different tissues. (c) Expression of *nka* in the gill of amphioxus cultured under 25‰ or 30‰ salinity. (d) Expression of *nkcc* in the gill of amphioxus cultured under 25‰ or 30‰ salinity. The *ef1α* was chosen as internal control for normalization. Data were from 3 independent experiments and expressed as mean ± SEM. The symbol ∗ shows *p* < 0.05.

Partial cloning revealed the presence of *nka* and *nkcc* in amphioxus that are highly identical to their counterparts in fish and mammalian species. Specifically, the *nka* cDNA fragment (GenBank accession number: KU312041) we cloned from *B. japonicum* was 629 bp long, encoding a deduced peptide of 209 amino acids which has 78.9% to 80.4% identity with vertebrate NKA3, and a cation_ATPase domain characteristic of NKA, and the *nkcc* cDNA fragment (GenBank accession number: KU312042) obtained was 960 bp long, coding for a deduced NKCC-like peptide of 319 amino acids, which possesses 45.8% to 48.3% identity with vertebrate NKCC1, and both AA_permease and SLC12 domains typical of NKCC (Supplementary Figures 2 and 3). As shown in Figures 2(a) and 2(b), both *nka* and *nkcc* displayed a tissue-specific expression, with relatively abundant levels in the gill. The gene *nkcc* was also abundantly expressed in the notochord. To test if salinity change affects the expression of *nka* and *nkcc* in the gill, the 25‰ salinity-acclimated animals were transferred to seawater with 30‰ salinity, and the expression of *nka* and *nkcc* in the gill was analyzed. No dead animals were observed. As shown in Figures 2(c) and 2(d), the transfer of 25‰ salinity-acclimated amphioxus to seawater with 30‰ salinity resulted in a significant upregulation of *nka* and *nkcc* in the gill. Compared with that of 25‰ group, the expression of *nka* in the gill of 30‰ group was increased to approximately 1.9-fold at 48 and 72 h after transfer. Similarly, compared with that of 25‰ group, the expression of *nkcc* in the gill of 30‰ group was increased to about 1.8-fold and 5.7-fold, respectively, at 48 and at 72 h after transfer. These suggested that NKA and NKCC were involved in the process of osmoregulation in the gill of amphioxus.

**Figure 3:**
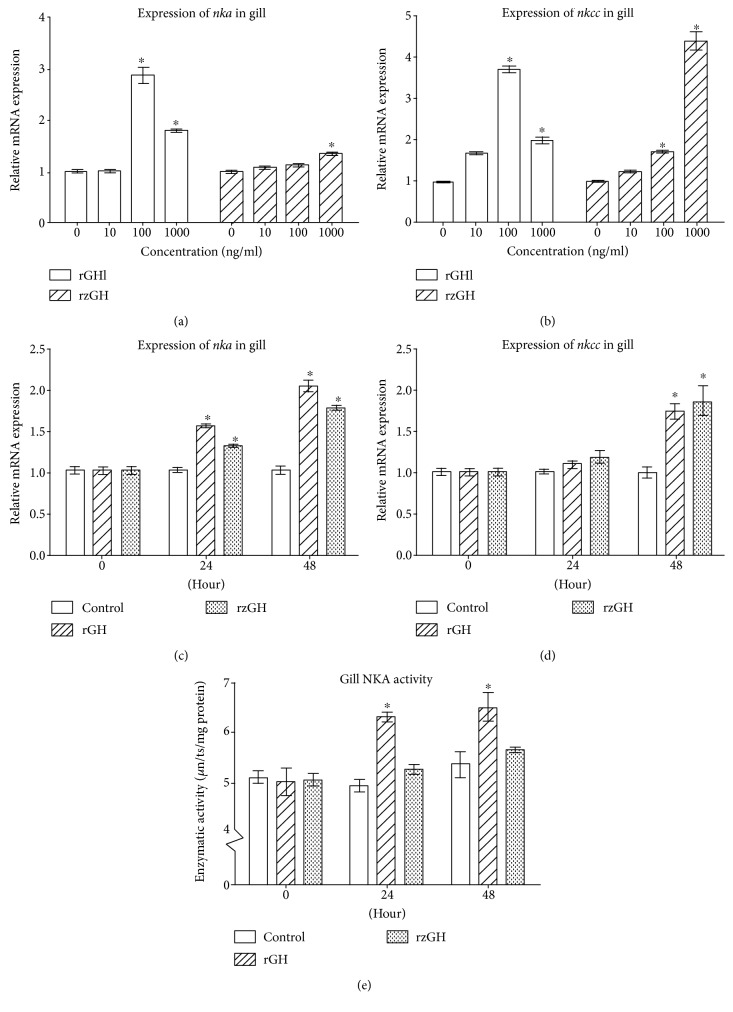
Induction of *nka* and *nkcc* expressions by rGHl. (a) Expression of *nka* in the gill cultures in response to rGHl or rzGH (concentrations ranging from 10 ng/ml to 1000 ng/ml). (b) Expression of *nkcc* in the gill cultures in response to rGHl or rzGH (concentrations ranging from 10 ng/ml to 1000 ng/ml). (c) Expression of *nka* in the gill of amphioxus injected with saline, rGHl, or rzGH. (d) Expression of *nkcc* in the gill of amphioxus injected with saline, rGHl, or rzGH. (e) NKA activity in the gill of amphioxus injected with saline, rGHl, or rzGH. One unit of enzymatic activity was defined as 1 *μ*moles ADP released per hour. Data were from 3 independent experiments and expressed as mean ± SEM. The symbol ∗ shows *p* < 0.05.

Next, we tested if rGHl can stimulate the expression of *nka* and *nkcc* in amphioxus gill. qRT-PCR showed that both rGHl and rzGH were capable of inducing an upregulation of *nka* and *nkcc* in a dose-dependent manner in the gill cultures. The expression of *nka* in the gill cultures was increased to about 1.8-fold after treatment with 1000 ng/ml rGHl (compared with control; the same below); similarly, the expression of the same gene in the gill cultures increased to about 1.4-fold after treatment with 1000 ng/ml rzGH. Moreover, the expression of *nkcc* in the gill cultures was increased to about 3.7-fold and 2.0-fold, respectively, after treatment with 100 and 1000 ng/ml rGHl, and its expression in the gill cultures was increased to about 1.7-fold and 4.4 fold, respectively, after treatment with 100 and 1000 ng/ml rzGH (Figures 3(a) and 3(b)). These indicated that GHl acted on the gill directly in a dose-dependent fashion in vitro for the first time, with 100 ng/ml concentration of rGHl being most effective in triggering the expression of *nka* and *nkcc*. Interestingly, incubation with 1000 ng/ml rGHl resulted in lowered expression of the genes. rzGH also displayed similar trend as rGHl. The reason for this trend is not clear at present. Possibly, it may be due to the failure of GHR dimerization caused by higher dose of rGHl or rzGH [33].

Injection of rGHl or rzGH into amphioxus also induced an increased expression of *nka* and *nkcc* in the gill in vivo. Compared with control group within the same time (the same below), the expression levels of *nka* in the gill were upregulated about 1.5-fold and 2.0-fold at 24 and 48 h after injection of rGHl, and the expression levels of the same gene upregulated about 1.3-fold and 1.8-fold at 24 and 48 h after injection of rzGH. Similarly, the expression of *nkcc* in the gill was about 1.8-fold higher than that of control at 48 h after injection of rGHl, and the expression of *nkcc* in the same tissue was about 1.9-fold higher than that of control at 48 h after injection of rzGH (Figures 3(c) and 3(d)). In addition, enzymatic activity assay revealed that injection of rGHl or rzGH into amphioxus triggered an increase in NKA activity in the gill at 24 to 48 h, consistent with the results of *nka* expression. The gill NKA activity in the gill of control group was 5.098 U/mg, 4.951 U/mg, and 5.363 U/mg, respectively, at 0 h, 24 h, and 48 h after injection. By contrast, the NKA activity in the gill of rGHl group was 5.028 U/mg, 6.304 U/mg, and 6.486 U/mg, individually, at 0 h, 24 h, and 48 h after injection, and the NKA activity in the gill of rzGH group was 5.044 U/mg, 5.259 U/mg, and 5.638 U/mg, separately, at 0 h, 24 h, and 48 h after injection (Figure 3(e)). Several studies have shown that injection of vertebrate GH induced about 1.5-fold to 2-fold increase in the NKA and NKCC in fish gill [11, 16, 28]. We demonstrated that amphioxus GHl, like vertebrate GH, was able to induce the expression of *nka* and *nkcc* in the gill in vivo. Besides, the trends of changes in the NKA activity and *nka* expression patterns affected by rGHl were both similar to that of rzGH. These showed that amphioxus GHl as well as zebrafish GH had a similar capacity to stimulate the expression of *nka* and *nkcc*, suggesting that GHl was able to mediate the salinity tolerance in amphioxus.

### 3.3. rGHl Increases Muscle Moisture

**Figure 4:**
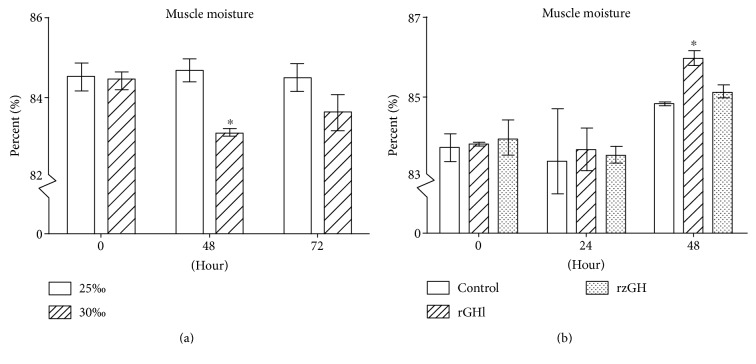
Muscle moisture of amphioxus. (a) Muscle moisture of amphioxus cultured under 25‰ or 30‰ salinity. (b) Muscle moisture of amphioxus injected with saline, rGHl, or rzGH. The animals were cultured in natural seawater. Data were from 3 independent experiments and expressed as mean ± SEM. The symbol ∗ shows *p* < 0.05.

Muscle usually loses moisture under high salinity environment. Thus, the relationship between salinity change and muscle moisture was tested. When the 25‰ salinity-acclimated animals were transferred to seawater with 30‰ salinity, their muscle moisture was 83.1% and 83.6% at 48 and 72 h after the transfer (Figure 4(a)), respectively. It is clear that the muscle moisture of the animals transferred to 30‰ salinity was significantly lower than that of 25‰ salinity-acclimated animals at 48 h after transfer. This indicated that higher salinity could reduce the muscle moisture of amphioxus in a short term.

Next, we tested the effect of rGHl on muscle moisture. When the animals cultured under 30‰ salinity were injected with saline, their muscle moisture was 83.4% and 84.8% at 24 and 48 h after injection (Figure 4(b)), respectively. By contrast, when the same animals were injected with saline plus rGHl or rzGH, their muscle moisture was increased to 83.7% and 86.0% as well as 83.5% and 85.1% at 24 and 48 h after injection (Figure 4(b)), individually. These indicated that rGHl as well as rzGH could increase the muscle moisture of amphioxus, providing an additional evidence that GHl was a regulator of salinity tolerance.

### 3.4. rGHl Induces Expression of *gh/prllbp*

**Figure 5:**
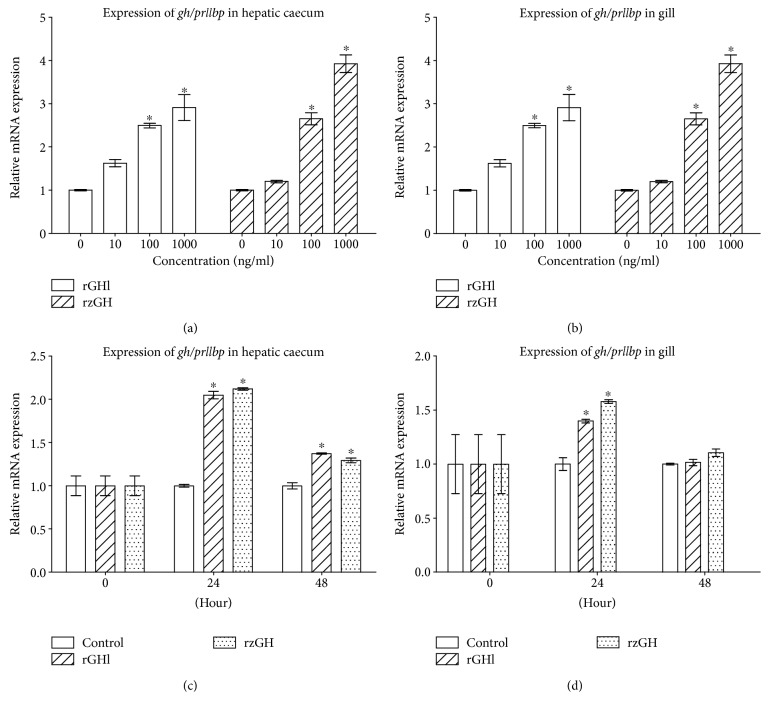
Induction of *gh/prllbp* expression by rGHl. (a) Expression of *gh/prllbp* in the cultures of the hepatic caecum in response to rGHl or rzGH (concentrations ranging from 10 ng/ml to 1000 ng/ml). (b) Expression of *gh/prllbp* in the cultures of the gill in response to rGHl or rzGH (concentrations ranging from 10 ng/ml to 1000 ng/ml). (c) Expression of *gh/prllbp* in the hepatic caecum of amphioxus injected with saline, rGHl, or rzGH. (d) Expression of *gh/prllbp* in the gill of amphioxus injected with saline, rGHl, or rzGH. Data were from 3 independent experiments and expressed as mean ± SEM. The symbol ∗ shows *p* < 0.05.

Sohm et al. [34] and Einarsdóttir et al. [35] both showed that GHBP was upregulated by GH and higher salinity in fish. To test if the GH/PRLlBP is associated with salinity tolerance of amphioxus, we examined the effects of rGHl and rzGH on the expression of *gh/prllbp* in the hepatic caecum and gill. As shown in Figures 5(a) and 5(b), both rGHl and rzGH clearly induced the expression of *gh/prllbp* in the tissue cultures of gill and hepatic caecum in a dose-dependent fashion (compared with control) which was similar to the results obtained by Sohm et al. [34] and Einarsdóttir et al. [35]. Similarly, injection of rGHl and rzGH stimulated the expression of *gh/prllbp* in the gill and hepatic caecum; and this increase in *gh/prlllbp* expression (Figures 5(c) and 5(d); compared with the control within the same time) was always related to higher concentration of rGHl or rzGH. These suggested that amphioxus GHl as well as zebrafish GH both could upregulate the expression of *gh/prllbp* in the gill and hepatic caecum, thereby contributing to salinity tolerance.

### 3.5. rGHl Induces Expression of *igfl*

**Figure 6:**
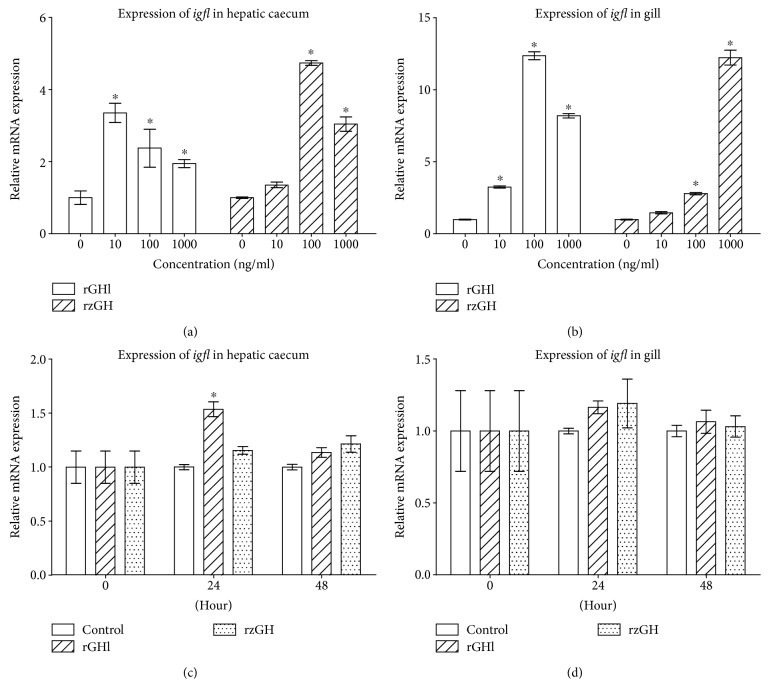
Induction of *igfl* expression by rGHl. (a) Expression of *igfl* in the cultures of the hepatic caecum in response to rGHl or rzGH (concentrations ranging from 10 ng/ml to 1000 ng/ml). (b) Expression of *igfl* in the cultures of the gill in response to rGHl or rzGH (concentrations ranging from 10 ng/ml to 1000 ng/ml). (c) Expression of *gh/prllbp* in the hepatic caecum of amphioxus injected with saline, rGHl, or rzGH. (d) Expression of *igfl* in the gill of amphioxus injected with saline, rGHl, or rzGH. Data were from 3 independent experiments and expressed as mean ± SEM. The symbol ∗ shows *p* < 0.05.

GHl was shown to be able to regulate growth through IGF action; thus, we tested the effects of rGHl and rzGH on the expression of *igfl* in the hepatic caecum and gill of amphioxus. As shown in Figures 6(a) and 6(b), both rGHl and rzGH induced the expression of *igfl* in the tissue cultures of hepatic caecum and gill in a dose-dependent fashion (compared with control). Moreover, injection of rGHl and rzGH stimulated the expression of *igfl* in the hepatic caecum (Figure 6(c); compared with control within the same time; the same below), though it had little effect on the expression of *igfl* in the gill (Figure 6(d)). The reason for this is not clear at present, but one possibility is that rGHl level transported to the gill by circulation might be rather lower because most rGHl had bound to its receptor in the hepatic caecum. Together, these data showed that amphioxus GHl could mediate salinity tolerance via stimulating the expression of *igfl*.

### 3.6. Salinity Increase Induces Expression of *ghl, gh/prllbp*, and *igfl*

**Figure 7:**
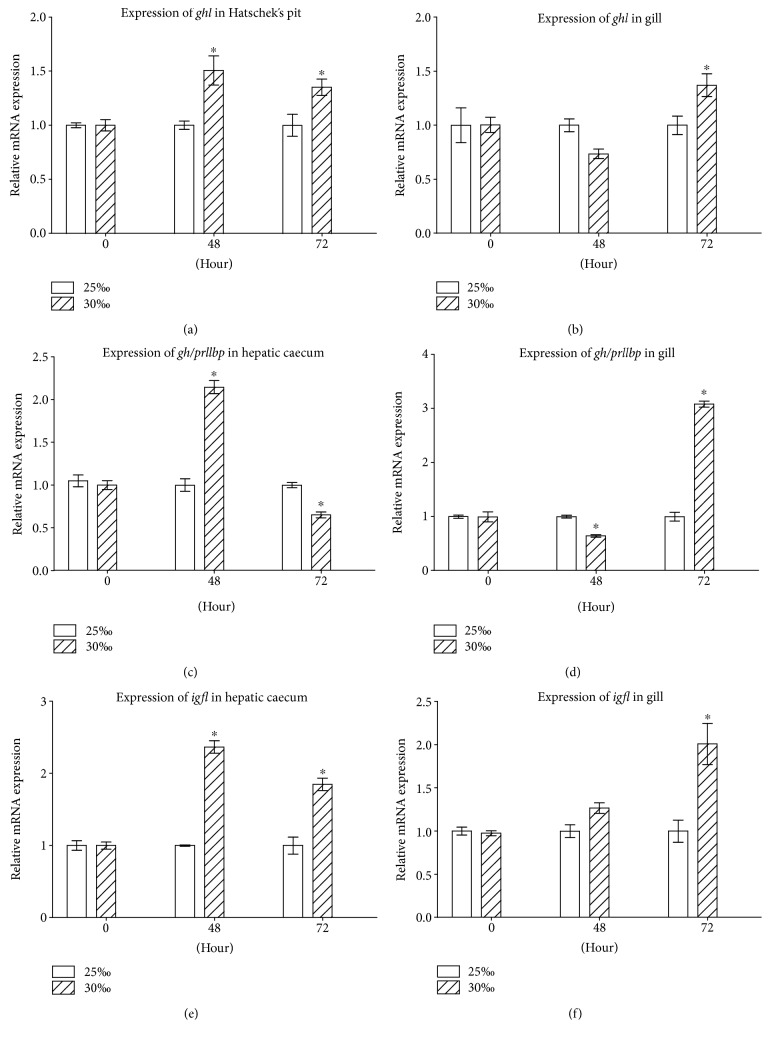
Expression of *ghl/igfl* axis genes of amphioxus cultured under 25‰ or 30‰ salinity. (a) Expression of *ghl* in the Hatschek's pit. (b) Expression of *ghl* in the gill. (c) Expression of *gh/prllbp* in the hepatic caecum. (d) Expression of *gh/prllbp* in the gill. (e) Expression of *igfl* in the hepatic caecum. (f) Expression of *igfl* in the gill. Data were from 3 independent experiments and expressed as mean ± SEM. The symbol ∗ shows *p* < 0.05.

To test if a vertebrate-like GH-IGF axis is involved in the osmoregulation in amphioxus, we transferred the 25‰ salinity-acclimated animals to seawater with 30‰ salinity and examined the effects of salinity change on the expression of *ghl* in the Hatschek's pit and gill as well as *gh/prllbp* and *igfl* in the hepatic caecum and gill. No dead animals were observed during the experimental periods. The transfer of 25‰ salinity-acclimated amphioxus to seawater with 30‰ salinity resulted in upregulation of *ghl* in the Hatschek's pit (Figure 7(a); compared with control within the same time; the same below) and gill (Figure 7(b)) as well as *igfl* in the hepatic caecum (Figure 7(e)) and gill (Figure 7(f)). The expression level of *gh/prllbp* in the hepatic caecum at 48 h after transfer (Figure 7(c)) well matched that of *gh* expression (Figure 7(a)). However, *gh/prllbp* expression in the tissue was soon decreased at 72 h after transfer (Figure 7(c)), suggesting that GH/PRLlBP may be also subjected to the downregulation by other factors independent of GHl [35]. Notably, as shown in Figure 7(a), *ghl* expression was upregulated at 48 h after transfer, but the expression of *igfl* in the gill at 48 h after transfer was not significantly elevated (Figure 7(f)). These indicated that the hepatic caecum was the primary target tissue of GHl. It should be noticed that even though there was little GHl binding in the gill, *nka* and *nkcc* in the gill were still significantly upregulated (Figures 2(c) and 2(d)). These suggested that IGFl, the mediator of GHl secreted by the hepatic caecum, may play a critical role in osmoregulation as IGF-I in fish [20, 21]. Together, all the data suggested that a vertebrate-like GHl/IGF system may be involved in osmoregulation in amphioxus.

## 4. Discussion

Functions of GH are diverse, with its growth-promoting and osmoregulatory activities being studied most extensively and intensively [4, 5, 7, 36]. Amphioxus GH-like protein GHl has been shown to have growth-promoting activity. Here, we demonstrate clearly that GHl has the capacity to mediate osmoregulation, as evidenced by the observations that GHl enhances not only survival rate of amphioxus but also muscle moisture under high salinity. In addition, we show that GHl induces upregulation of both the ion transporter NKA and cotransporter NKCC in the gill as well as mediator of GH action IGFl in the hepatic caecum, indicating that GHl apparently fulfills this osmoregulatory activity through the same mechanisms of vertebrate GH. Collectively, these data suggest that osmoregulatory activities of GH already emerged in the basal chordate [37], much earlier than thought before [38].

Teleosts are well adapted for ion exchange using active and passive mechanisms across various surface membranes to keep the osmotic pressure of their body fluids steady when they face relatively hyperosmotic or hypoosmotic environment [39]. Interestingly, Huang [40] has reported that the average osmotic pressure of amphioxus body fluids was about 780 mOsm/L, which was significantly lower than that of ambient seawater, 1200 mOsm/L, suggesting that amphioxus may be able to transport ions across surface membranes to achieve proper osmotic balance and hydration status. However, this demands further study.

The gill, in addition to being a respiratory organ, is the primary site of net sodium and chloride transport in fishes, and the roles of chloride cells in the gill are well accepted as the principal site of ion extrusion in seawater or in a hypertonic environment [18, 41]. The ciliated gill bars of amphioxus are radically different from fish gill structure, and if they function in respiration remains controversial [42–44]. We show that GHl is able to induce the expression of both *nka* and *nkcc* as well as *gh/prllbp* in the gill of amphioxus, suggesting that the gill may also be the main osmoregulatory organ in amphioxus.

**Figure 8:**
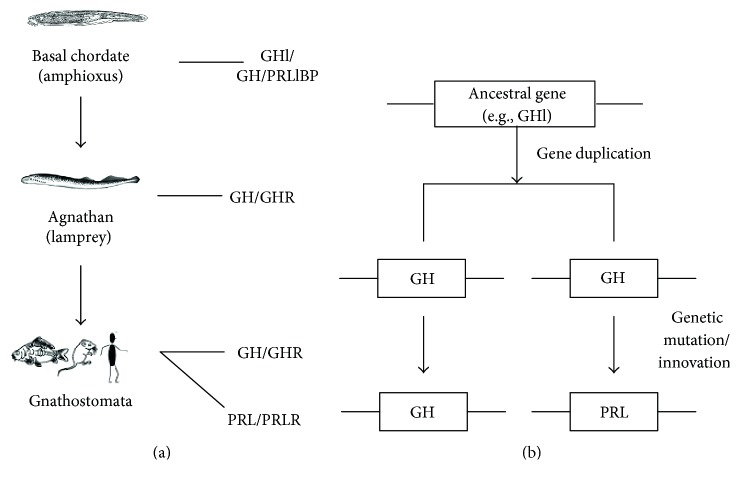
A proposed model for the evolution of GH/PRL family. (a) Ancestral GH/GHR system already emerged in the basal chordate, and PRL/PRLR originated with the advent of Gnathostomata. (b) The ancestral gene, like amphioxus GHl gene, generated two genes by gene duplication early in vertebrate evolution. One of the resulting genes evolved directly into GH gene in modern Gnathostomata, while the other gene evolved into PRL gene, possibly due to adaptation of FW habitats, by genetic innovations and/or mutation after split of agnathan/Gnathostomata.

GHl has been shown to be the only member of the vertebrate pituitary hormone family in amphioxus, which is capable of promoting growth [26]. Here, we show that GHl is involved in the mediation of osmoregulation. It is apparent that GHl plays a dual role of both growth promotion and osmoregulation. These allow us to propose that the function of the ancestral gene that contributed to the origin of GH/PRL family was to regulate not only somatic growth (i.e., GH-like) but also hydrominal balance (i.e., PRL-like). Thus, the ancestral gene, like amphioxus *ghl*, might generate two genes by gene duplication early in vertebrate evolution. One of the resulting genes evolved directly into GH gene in modern gnathans, while the other gene evolved into PRL gene, possibly due to adaptation of FW habitats, by genetic innovations and/or mutation after split of agnathans/gnathans (Figure 8). Previously, two models have been suggested to depict the origin of GH/PRL family. The first model was developed on the basis of the examination of vertebrate history and the study of ancient chordates and suggests that the primary function of the ancestral gene that gave rise to the GH/PRL family was GH-like; that is, its original activity was to regulate somatic growth, while PRL activity evolved later, perhaps to allow for the colonization of FW habitats. The opposing view claims that the ancestral gene was involved in osmoregulation, because this one function is common to fish PRL and GH [45]. It is obvious that the two old models become unified in our new model. Of note, only GH but not PRL have been detected in lamprey [46, 47]. Thus, the growth-promoting and osmoregulatory properties of the single hormone remained unchanged in agnathans. Further comparative and functional studies of GH and PRL will shed more light on the divergence and development of these structurally and functionally related hormones and their receptors.

In conclusion, this study highlights amphioxus GHl, in addition to growth-promoting activity, which can mediate osmoregulation through the same mechanisms of vertebrate GH. It also proposes a new model depicting the origin of GH/PRL family in vertebrates.

## Supplementary Material

Supplementary Material contains the SDS-PAGE and Western-blotting of rzPRL, sequence information and analysis.

## Figures and Tables

**Table 1:** Sequences of the primers used in this study.

Gene	Primer	Sequence (5′ to 3′)	Sequence information
*Zebrafish prl*	P1 (sense)	GAAACCTGTTCTAGTAATGGCTCAAG	cDNA primer
	P2 (antisense)	CTGCGGTCAGAACTTACCTTAGAAT	
	P3 (sense)	CCGGAATTCGTGGGTCTGAATGATTTG	Recombinant primer
	P4 (antisense)	CCGCTCGAGCTAGCACATGTCAGGCC	
*Amphioxus nka*	P5 (sense)	GCTGGCTACAGTGACGGTATGTC	cDNA primer
	P6 (antisense)	CCAGTTCCAGGTAGGCGTTGT	
	P7 (sense)	TGCGTAACTTGGAGGCTGTGGAAA	Real-time PCR primer
	P8 (antisense)	GCCAGGTTGGGTTGCTCTTGTCATA	
*Amphioxus nkcc*	P9 (sense)	CGCCATCGCTCCTCTCATCTCT	cDNA primer
	P10 (antisense)	CTCGGTAGGTCACTTGCTATTGTCAG	
	P11 (sense)	TGACTGGGATGGGTAAGATGAGGC	Real-time PCR primer
	P12 (antisense)	TGGTCATTCTGTCTTTCTTGGTCTCG	
*Amphioxus ef1α*	P13 (sense)	TGCTGATTGTGGCTGCTGGTACTG	Real-time PCR primer
	P14 (antisense)	GGTGTAGGCCAGCAGGGCGTG	
*Amphioxus gh/prllbp*	P15 (sense)	GAAGACTCGGACCTGGAGACGCACTA	Real-time PCR primer
	P16 (antisense)	CGTGTTCAGGTAGGCGTGGTCGTA	
*Amphioxus igfl*	P17 (sense)	CTCATCCGCCCATCAGTA	Real-time PCR primer
	P18 (antisense)	GGTTCTTTCTTGTCCGTTT	
*Amphioxus ghl*	P19 (sense)	CGCTGTTCTTAGACGAGGTTTTGCT	Real-time PCR primer
	P20 (antisense)	CGGTGATGTCAGTAGGCTGGGTG	

## References

[1] Boulay J., O’Shea J., Paul W. (2003). Molecular phylogeny within type I cytokines and their cognate receptors. *Immunity*.

[2] Huising M., Kruiswijk C., Flik G. (2006). Phylogeny and evolution of class-I helical cytokines. *Journal of Endocrinology*.

[3] Ono M., Takayama Y., Rand-Weaver M. (1990). cDNA cloning of somatolactin, a pituitary protein related to growth hormone and prolactin. *Proceedings of the National Academy of Sciences*.

[4] Forsyth I., Wallis M. (2002). Growth hormone and prolactin—molecular and functional evolution. *Journal of Mammary Gland Biology and Neoplasia*.

[5] Horseman N. D., Yu-Lee L. Y. (1994). Transcriptional regulation by the helix bundle peptide hormones: growth hormone, prolactin, and hematopoietic cytokines. *Endocrine Reviews*.

[6] McCormick S. D. (2001). Endocrine control of osmoregulation in teleost. *Washington American Zoologist*.

[7] Sakamoto T., McCormick S. D. (2006). Prolactin and growth hormone in fish osmoregulation. *General and Comparative Endocrinology*.

[8] Sakamoto T., Mccormick S. D., Hirano T. (1993). Osmoregulatory actions of growth hormone and its mode of action in salmonids: a review. *Fish Physiology and Biochemistry*.

[9] Auperin B., Leguen I., Rentier-Delrue F., Smal J., Prunet P. (1995). Absence of a tiGH effect on adaptability to brackish water in tilapia (*Oreochromis niloticus*). *General and Comparative Endocrinology*.

[10] Xu B., Miao H., Zhang P., Li D. (1997). Osmoregulatory actions of growth hormone in juvenile tilapia (*Oreochromis niloticus*). *Fish Physiology and Biochemistry*.

[11] Mancera J. M., McCormick S. D. (1998). Evidence for growth hormone/insulin-like growth factor I axis regulation of seawater acclimation in the euryhaline teleost *Fundulus heteroclitus*. *General and Comparative Endocrinology*.

[12] Pickford G. E., Phillips J. G. (1959). Prolactin, a factor in promoting survival of hypophysectomized killifish in fresh water. *Science*.

[13] Dharmamba M., Maetz J. (1972). Effects of hypophysectomy and prolactin on the sodium balance of *Tilapia mossambica* in fresh water. *General and Comparative Endocrinology*.

[14] Madsen S. S., Jensen M. K., Nohr J., Kristiansen K. (1995). Expression of Na^+^-K^+^-ATPase in the brown trout, *Salmo trutta*: in vivo modulation by hormones and seawater. *American Journal of Physiology-Regulatory, Integrative and Comparative Physiology*.

[15] Sakamoto T., Shepherd B. S., Madsen S. S. (1997). Osmoregulatory actions of growth hormone and prolactin in an advanced teleost. *General and Comparative Endocrinology*.

[16] Pelis R., McCormick S. D. (2001). Effects of growth hormone and cortisol on Na^+^-K^+^-2Cl^−^ co-transporter localization and abundance in the gills of Atlantic salmon. *General and Comparative Endocrinology*.

[17] Silva P., Solomon R., Spokes K., Epstein F. (1977). Ouabain inhibition of gill Na^+^-K^+^-ATPase: relationship to active chloride transport. *Journal of Experimental Zoology*.

[18] Degnan K. J. (1984). Chloride secretion by teleost gill and operculum. *Chloride Transport Coupling in Biological Membranes and Epithelia*.

[19] Tang C. H., Lee T. H. (2007). The effect of environmental salinity on the protein expression of Na^+^/K^+^-ATPase, Na^+^/K^+^/2Cl^−^ cotransporter, cystic fibrosis transmembrane conductance regulator, anion exchanger 1, and chloride channel 3 in gills of a euryhaline teleost, *Tetraodon nigroviridis*. *Comparative Biochemistry and Physiology. Part A, Molecular & Integrative Physiology*.

[20] Madsen S. S., Bern H. A. (1993). In-vitro effects of insulin-like growth factor-I on gill Na^+^,K^+^-ATPase in coho salmon, *Oncorhynchus kisutch*. *Journal of Endocrinology*.

[21] Duan C. (1997). The insulin-like growth factor system and its biological actions in fish. *American Zoologist*.

[22] Hirano T. (1986). The spectrum of prolactin action in teleosts. *Progress in Clinical and Biological Research*.

[23] Manzon L. A. (2002). The role of prolactin in fish osmoregulation: a review. *General and Comparative Endocrinology*.

[24] Breves J. P., Mccormick S. D., Karlstrom R. O. (2014). Prolactin and teleost ionocytes: new insights into cellular and molecular targets of prolactin in vertebrate epithelia. *General and Comparative Endocrinology*.

[25] Takei Y., Hiroi J., Takahashi H., Sakamoto T. (2014). Diverse mechanisms for body fluid regulation in teleost fishes. *American Journal of Physiology—Regulatory, Integrative and Comparative Physiology*.

[26] Li M., Gao Z., Ji D., Zhang S. (2014). Functional characterization of GH-like homolog in amphioxus reveals an ancient origin of GH/GH receptor system. *Endocrinology*.

[27] Wang Y., Zhang S. (2011). Expression and regulation by thyroid hormone (TH) of zebrafish IGF-I gene and amphioxus IGFl gene with implication of the origin of TH/IGF signaling pathway. *Comparative Biochemistry and Physiology. Part A, Molecular & Integrative Physiology*.

[28] Mccormick S. D. (1996). Effects of growth hormone and insulin-like growth factor I on salinity tolerance and gill Na^+^, K^+^-ATPase in Atlantic salmon (*Salmo salar*): interaction with cortisol. *General and Comparative Endocrinology*.

[29] Lalitha S. (2000). Primer premier 5. *Biotech Software & Internet Report: The Computer Software Journal for Scient*.

[30] Fang Y. Q., Wang L. (1984). The preliminary study of homogenate of the wheel organ and Hatschek’s pit of amphioxus on testicular development in young toad (Bufo malanostictus). *Acta Biologiae Experimentalis Sinica*.

[31] Breves J. P., Serizier S. B., Goffin V., McCormick S. D., Karistrom R. O. (2013). Prolactin regulates transcription of the ion uptake Na^+^/Cl^−^ cotransporter (ncc) gene in zebrafish gill. *Molecular and Cellular Endocrinology*.

[32] Zeng L., Lei J. L., Liu B., Hong W. S., Ai C. X., Gao C. R. (2014). Effects of salinity on Na^+^-K^+^-ATPase activity in gills, and concentrations of ions and hormones in serum of juvenile turbot (*Scophthalmus maximus*). *Chinese Journal of Zoology*.

[33] Frank S. J., Messina J. L. (2002). *Growth hormone receptor, cytokine reference online*.

[34] Sohm F., Manfroid I., Pezet A. (1998). Identification and modulation of a growth hormone-binding protein in rainbow trout (*Oncorhynchus mykiss*) plasma during seawater adaptation. *General and Comparative Endocrinology*.

[35] Einarsdóttir I. E., Gong N., Jönsson E. (2014). Plasma growth hormone-binding protein levels in Atlantic salmon *Salmo salar* during smoltification and seawater transfer. *Journal of Fish Biology*.

[36] Bern H. A. (1983). Functional evolution of prolactin and growth hormone in lower vertebrates. *American Zoologist*.

[37] Putnam N., Butts T., Ferrier D. E. (2008). The amphioxus genome and the evolution of the chordate karyotype. *Nature*.

[38] Specker J. L., Ingleton P. M., Bern H. A. (1984). *Comparative physiology of the prolactin cell. Prolactin secretion: a multidisciplinary approach*.

[39] Greenwell M. G., Sherrill J., Clayton L.A. (2003). Osmoregulation in fish. *The Veterinary Clinics of North America Exotic Animal Practice*.

[40] Huang S. F. (2007). *Comparative-genomic analysis of the amphioxus immune system and functional study of its TLR and TNF signal pathways, Doctoral dissertation*.

[41] Pisam M. (1981). Membranous systems in the “chloride cell” of teleostean fish gill; their modifications in response to the salinity of the environment. *Anatomical Record*.

[42] Kent G. C., Miller L. (1997). *Comparative Anatomy of Vertebrates*.

[43] Storch V., Welsch U. (1999). *Kükenthal- Zoologisches Praktikum*.

[44] Schmitz A., Gemmel M., Perry S. F. (2000). Morphometric partitioning of respiratory surfaces in amphioxus (*Branchiostoma lanceolatum* Pallas). *Journal of Experimental Biology*.

[45] Chen T. T. (1994). Structure and evolution of fish growth hormone and insulin like growth factor genes. *Fish Physiology*.

[46] Kawauchi H., Suzuki K., Yamazaki T. (2002). Identification of growth hormone in the sea lamprey, an extant representative of a group of the most ancient vertebrates. *Endocrinology*.

[47] Ellens E. R., Kittilson J. D., Hall J. A., Sower S. A., Sheridan M. A. (2013). Evolutionary origin and divergence of the growth hormone receptor family: insight from studies on sea lamprey. *General and Comparative Endocrinology*.

