# Supplementary material for: Activities of Amphioxus GH-Like Protein in Osmoregulation: Insight into Origin of Vertebrate GH Family
Source: Int J Endocrinol. 2017 Mar 17;2017:9538685. doi: 10.1155/2017/9538685 (PMC5376476; doi:10.1155/2017/9538685)
Supplement: Supplementary file 1 — Supplementary Material contains the SDS-PAGE and Western-blotting of rzPRL, sequence information and analysis. [file 9538685.f1.doc]

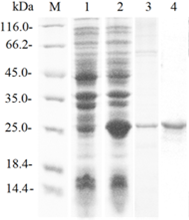


**Supplementary Fig. 1.** **SDS-PAGE and Western blotting of recombinant protein rzPRL.** M. markers; Lane 1, total cellular extract from *E. coli* BL21 containing *prl*-inserted expression vector before induction; lane 2, total cellular extract from IPTG-induced *E. coli* BL21 containing *prl*-inserted expression vectors; lane 3, purified rzPRL; lane 4, Western blot of corresponding purified rzPRL, which was immunostained with anti-His-tag antibody.


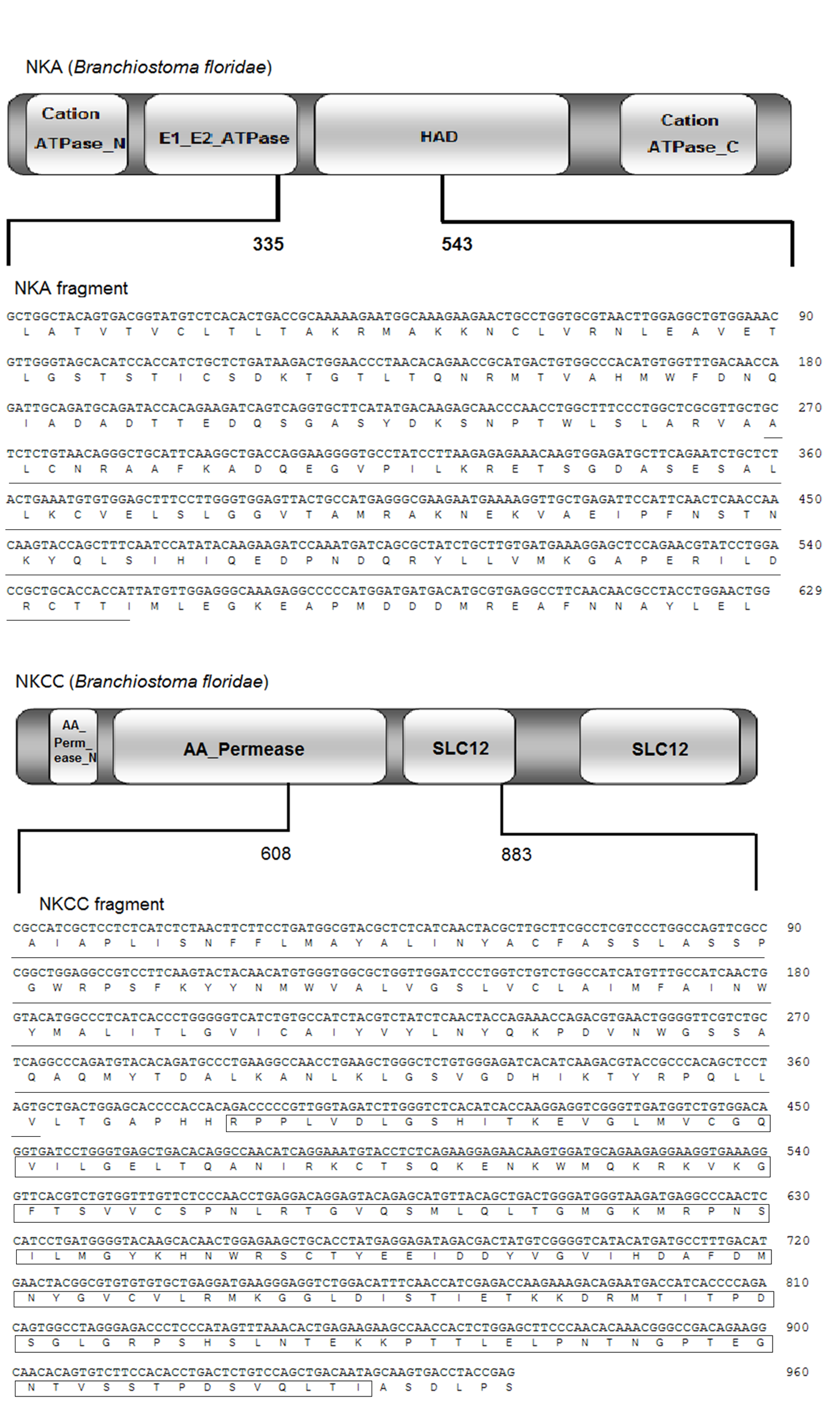


**Supplementary Fig. 2. Nucleotide and deduced amino acid sequences of *nka* and *nkcc* fragments.** The cation_ATPase domain of NKA and AA_Permease domain of NKCC were underlined; the SLC12 domain of NKCC was marked by open box.


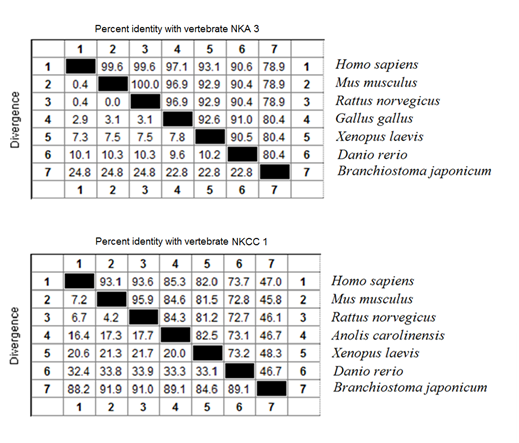


**Supplementary Fig. 3.** Amino acid sequence comparison of NKA and NKCC. Accession numbers of the sequences used in comparison are: human NKA3 (NP_689509.1); mouse NKA3 (NP_001277398.1); rat NKA3 (NP_036638.1); chicken NKA3 (NP_990806.1); frog NKA3 (NP_001120366.1); zebrafish NKA3 (NP_571759.2); human NKCC1 (NP_001037.1); mouse NKCC1 (AAC77832.1); rat NKCC1 (NP_113986.1); lizard NKCC1 (XP_003216468.1); frog NKCC1 (XP_002931755.1); zebrafish NKCC1 (NP_001157126.1).
